# Does a functional activity programme improve function, quality of life, and falls for residents in long term care? Cluster randomised controlled trial

**DOI:** 10.1136/bmj.a1445

**Published:** 2008-10-09

**Authors:** Ngaire Kerse, Kathy Peri, Elizabeth Robinson, Tim Wilkinson, Martin von Randow, Liz Kiata, John Parsons, Nancy Latham, Matthew Parsons, Jane Willingale, Paul Brown, Bruce Arroll

**Affiliations:** 1 Department of General Practice and Primary Health Care, School of Population Health, University of Auckland, Private Bag 92019, Auckland, 1001, New Zealand; 2 School of Nursing, University of Auckland; 3 Department of Epidemiology and Biostatistics, School of Population Health, University of Auckland; 4 Health Care of the Elderly, University of Otago, Christchurch, New Zealand; 5 Department of Sociology, University of Auckland; 6 Health and Disability Research Unit, Boston University School of Public Health, Boston, MA, USA; 7 Health Systems, School of Population Health, University of Auckland

## Abstract

**Objective** To assess the effectiveness of an activity programme in improving function, quality of life, and falls in older people in residential care.

**Design** Cluster randomised controlled trial with one year follow-up.

**Setting** 41 low level dependency residential care homes in New Zealand.

**Participants** 682 people aged 65 years or over.

**Interventions** 330 residents were offered a goal setting and individualised activities of daily living activity programme by a gerontology nurse, reinforced by usual healthcare assistants; 352 residents received social visits.

**Main outcome measures** Function (late life function and disability instruments, elderly mobility scale, FICSIT-4 balance test, timed up and go test), quality of life (life satisfaction index, EuroQol), and falls (time to fall over 12 months). Secondary outcomes were depressive symptoms and hospital admissions.

**Results** 473 (70%) participants completed the trial. The programme had no impact overall. However, in contrast to residents with impaired cognition (no differences between intervention and control group), those with normal cognition in the intervention group may have maintained overall function (late life function and disability instrument total function, P=0.024) and lower limb function (late life function and disability instrument basic lower extremity, P=0.015). In residents with cognitive impairment, the likelihood of depression increased in the intervention group. No other outcomes differed between groups.

**Conclusion** A programme of functional rehabilitation had minimal impact for elderly people in residential care with normal cognition but was not beneficial for those with poor cognition.

**Trial registration** Australian Clinical Trials Register ACTRN12605000667617.

## Introduction

Elderly people with high levels of habitual physical activity live longer and have better general health and higher levels of wellbeing than do those who are inactive.^1^ Improving activity levels may improve health outcomes^2^ ^3^ ^4^; however, for very frail elderly people in residential care, the potential for such activity is limited. Even small gains in functional status may result in significant benefits in functional performance and quality of life.^4^ Although some success in slowing functional decline has been shown,^4^ ^5^ sustainable ways to do this in residential care have not yet been identified. The challenges are to design an intervention that is acceptable to and will involve a large proportion of older residents.

Successful approaches in residential care have included programmes designed to prevent falls,^6^ progressive resistance training,^7^ nutrition programmes, and seated activities.^8^ Use of weights or elastic bands can improve strength and slow decline in activities of daily living functions.^9^ Such successful programmes have involved a combination of staff and visiting exercise specialists.

Older muscles can be retrained and become stronger with repetitive use,^10^ but activity must be sustained to improve functional status.^11^ Low intensity activity is a reasonable goal when incorporated into daily activities,^12^ as it is more acceptable to elderly people than vigorous activity and has greater potential for long term compliance.^13^ An individualised programme of progressive repetitions of activities of daily living increases the ability to perform these independently,^14^ and this is a potential way to encourage moderate exercise. However, to ensure participation, supervision and encouragement from usual attendants are likely to be needed.

Activity programmes must be safe to be effective. Some trials have suggested that improving function and mobility in frail elderly people increases their risk of falls and related injuries.^15^ ^16^ ^17^ Fall rates in residential care are three times those of elderly people living in the community,^18^ and hip fractures are 10.5 times more likely than for community dwellers.^19^ Only 15% of those who fracture their hip regain their pre-injury functional level, placing a large burden of care on staff.^20^ This means that falls are a relevant outcome in activity trials involving frail elderly people.

We report the results of a pragmatic cluster randomised controlled trial in a representative sample of residential care homes. This follows a successful efficacy trial, which suggested that an intervention based on activities that are meaningful to the individual person can be effective in improving quality of life for elderly people in residential care.^21^

## Methods

A cluster randomised controlled trial tested the effect of an activity programme in a residential care population on functionality, quality of life, and falls over 12 months. The trial is reported according to the CONSORT statement.^22^

### Participants and recruitment

Further recruitment details are reported elsewhere.^23^ Residents in low level dependency residential care in two cities of New Zealand were eligible for this study. Elderly people living in low level dependency residential care homes need assistance with most instrumental activities of daily living and at least two activities of daily living but can usually ambulate to some degree and feed themselves.

The New Zealand Ministry of Health supplied a listing of all residential care homes (subsequently termed “homes”) in the two centres. We excluded homes caring exclusively for young disabled patients or delivering only palliative care. We invited homes to participate in random order by using computer generated random numbers. The owners and managers of the homes and all eligible residents, their family members, or guardians gave written informed consent.

Eligible residents were aged 65 years and over, able to engage in a conversation about a goal, remember the goal, and participate in a programme to achieve the goal (a proxy for cognitive state). The clinical nurse in charge of the resident at the time of recruitment judged ability. We excluded residents who were unable to communicate to complete the study measures, had anxiety as their main diagnosis, were acutely unwell, or were in a terminal state. We recruited residents between February and November 2004 and followed each resident for 12 months.

### Measures

We ascertained staffing levels—registered nurses’ hours per day and healthcare assistants’ hours per day—by a structured interview of management staff. Trained independent research nurses recruited residents and, using standardised techniques, collected demographic data, health information, and drug use data from the medical and nursing record and did standardised face to face interviews. We estimated socioeconomic status by using the main occupation of the person or their spouse and by establishing whether the resident was publicly funded by a means tested entitlement. We used Hodgkinson’s abbreviated mental test score to establish cognitive function.^24^

The primary outcomes were function, self reported and observed; quality of life; and falls over 12 months of follow-up. We measured self reported function with the late life function and disability instrument validated for frail elderly people.^25^ The two main components, the functional and disability components, were administered at baseline and 12 months’ follow-up. Sub-domains included upper extremity function, basic lower extremity function, and advanced lower extremity function. The function component was also administered at six months.

We assessed observed basic mobility and functional tasks at baseline, six months, and 12 months with the timed up and go,^26^ the elderly mobility scale,^27^ and the FICSIT-4 balance test.^28^ We measured quality of life at all three time points with the EuroQol instrument and the life satisfaction index.^29^ ^30^ We defined falls as “an unexpected event in which the participants come to rest on the ground, floor, or other lower level.”^31^ Independent researchers used this definition to audit all medical and nursing records of participating residents for three months before the trial started and then every two months over the year of the trial.

Secondary outcome measures were depressive symptoms assessed by the geriatric depression scale,^32^ fear of falling measured by the modified fear of falling scale,^33^ and hospital admissions over the 12 months of the trial from the national minimum dataset, which records all discharges from New Zealand’s public hospitals and is held by the New Zealand Health Information Service.

We ascertained adverse events, including muscle pain, fatigue, and general aches and pains, by self report at baseline, six months, and 12 months. We established mortality and permanent admission to a high dependency level of long term care (nursing home care) by direct enquiry at each follow-up visit.

### Randomisation and blinding

After recruitment of all homes and residents and collection of baseline data, a biostatistician not involved in recruitment randomised homes to the intervention or control group by using computer generated random numbers. We used randomisation by home (cluster) to avoid contamination between groups resulting from the intervention design involving staff training and thus potentially affecting all residents in the home.^34^ Research nurses blinded to the group allocation of the homes used standardised methods to assess outcomes. All intervention materials were removed from each resident’s room before follow-up reassessment visits.

### Interventions

Two trained gerontology nurses, one at each site, delivered the promoting independence in residential care (PIRC) intervention on the basis of a successful efficacy trial.^21^ To standardise intervention delivery they were trained by KP and did dual assessments and plan development for five residents at the start, after six months, and towards the end of the enrolment period at both sites. The gerontology nurses were not involved in any of the outcome assessments and had a well designed study protocol to guide the intervention phases.

*Goal setting*—The resident, assisted by the gerontology nurse, set a mutually agreed goal that had to meet two criteria: it had to be relevant and meaningful to the resident, and it had to promote progressive increases in physical activity. The goal setting often required one to two visits, depending on the resident’s abilities

*Functional assessment and activity programme design*—The gerontology nurse then completed a functional assessment and designed an individualised programme of physical activities based on repetitions of activities of daily living, such as rising from a chair, additional walking, or repeated transfers, aiming to improve the physical functions needed to achieve the goal. Exercise activities were planned to be done daily or several times a day in short doses as part of the resident’s usual activities. A physiotherapist and occupational therapist were available to assist in assessment and programme design when asked by the gerontology nurse. A prescriptive plan, the promoting independence plan, was constructed and placed on the wall in the resident’s room and in the resident’s file. The plan was developed in part as a template, such that if rising from a chair was challenging (and was needed as part of achieving the goal) the plan incorporated initially five sit to stands twice daily with increasing repetitions as the individual resident gained greater lower leg strength.

*Staff implementation*—The gerontology nurse trained the healthcare assistants in implementing the promoting independence plan on a one to one basis for each resident. The healthcare assistants then implemented the plan under the supervision of the usual nursing staff of the facility. For the more independent residents, the healthcare assistants provided minimal supervision. The more dependent residents needed up to 15 minutes of close supervision of the plan twice daily.

*Ongoing support*—The gerontology nurse provided support to the home staff every week for the first month and monthly for the next six months. Support included visits; discussions about residents, exercises, and goals; and reviewing progress and renegotiating a new goal with enrolled residents when the first goal was met. The intervention lasted six months in total. After that time the facility staff were expected to continue encouraging engagement in activity.

*Control group*—Residents in control group homes received usual care and were offered two social visits by a social science researcher to control for the attention received by the resident from the gerontology nurse visits.

*Uptake*—Gerontology intervention nurses following enrolled residents reported compliance of residents with the activity programme. Staff were asked to complete checklists of residents’ episodes of exercises. A record was made of all social visits.

### Sample size calculation

We used previous data on the main outcome variables to complete power calculations. Based on a baseline average score of 15 (SD 6) of the elderly mobility scale,^35^ we needed 132 participants in each group to detect a true difference of 2 units (90% power, 0.05 level of significance, two sided test). Inflating this according to an estimated design effect of 1.44 to account for the cluster randomised design (intraclass correlation of 0.02 from the efficacy study^21^), we needed 190 residents in each group. To detect a 20% reduction in falls and a 15% reduction in injuries with 90% power at the 0.05 level of significance (two sided test), we needed 329 residents for falls and 235 residents for injuries, based on a baseline proportion of 46% falling and 30% sustaining injury over a year and inflating raw estimates to allow a design effect of 3.2 for falls and 1.8 for injuries.^16^ ^36^ Allowing a response rate of 93%,^16^ and taking the largest of these estimates, we needed a total of 707 elderly people to participate. These were to be recruited from between 40 and 44 homes, assuming an average cluster size of 20 based on half the residents in homes being eligible and the average home size being 40 people.^21^

### Statistical analysis

We sought to examine the effect of the intervention at an individual level, adjusting for the impact on variance of the clustering inherent in the sampling design process. We investigated differences in changes over time in the two groups by testing the effect of the interaction of time with the randomisation group on the main outcomes. We did planned tests for interactions between depression, randomisation group, and time and between mental status, intervention group, and time. If appropriate, we did subgroup analyses.

We described baseline characteristics for intervention and control groups. We used generalised linear mixed models, with rest home as a random factor, city as a fixed factor, and time as a repeated measure, to investigate whether the intervention was associated with a change in function (late life function and disability index, timed up and go, elderly mobility scale, balance) or quality of life (life satisfaction index, EuroQol) over time and in particular whether this change differed between the social and intervention groups. We used an autoregressive structure to model the correlations between repeated measures over time. Where outcome measures were not normally distributed, we log transformed them or converted them to categorical variables for analysis. Where appropriate, we used a logit link for binary data. We used a generalised linear mixed model with a logit link for the secondary outcome of depression. We investigated time to fall, allowing for multiple falls per resident, by using a Cox proportional hazards model with rest home included as a random effect.

We included mental status (abbreviated mental test score), depression (geriatric depression scale score), age, sex, previous falls, total number of drugs (as a proxy for health status), time in the low level dependency home, and socioeconomic status in the models for the primary outcomes. We used negative binomial regression models allowing for the clustering of participants within rest homes to investigate hospital admissions and adverse effects (aches and pains). We used the χ^2^ test to test for differences in the proportion of participants who were admitted to high level dependency care or died. We used SAS version 9.1 for all analyses.

The quality of the trial met all criteria outlined for randomised controlled trials in the *User’s Guide to the Medical Literature II*,^37^ except that double blinding was not possible. 

## Results

Forty one of 46 randomly selected homes participated; 83% of eligible residents participated (330 activity group, 352 social group). Staffing levels were similar for registered nurses (P=0.24) and healthcare assistant staff (P=0.40), and recruitment rates were similar between intervention and control homes. The figure[Fig fig1] shows the flow of residents through the trial. All homes recruited completed the trial. The mean age of residents was 84 (SD 7) years, and 504 (74%) residents were women. Characteristics of residents were mainly evenly balanced between the groups (table 1). In seven of the 41 homes, a staff member or resident unblinded the assessor at some time during follow-up. This potentially affected measures on 56 activity participants and 41 social participants.

**Figure fig1:**
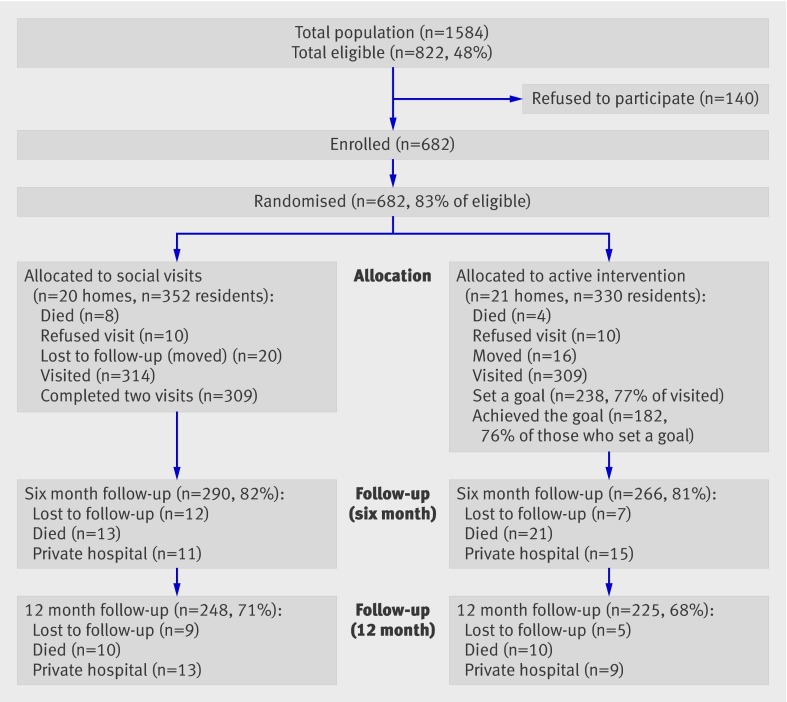
Flow of participants through trial

**Table 1 tbl1:** Characteristics and outcome measures at baseline for all study participants. Values are mean (SD) unless stated otherwise

Characteristic	Social group (n=352)	Activity group (n=330)	Total (n=682)
Participants enrolled per rest home	16.8 (9.4)	16.5 (10.8)	16.5 (10.0)
Response rate (%)	14 (81)	12 (84)	13 (83)
Nursing assistant hours/resident/day	1.41 (0.49)	1.52 (0.35	1.47 (0.43)
Registered nurse hours/resident/day	0.28 (0.12)	0.34 (0.20)	0.30 (0.17)
Level of care:			
Support needs assessment	3.22 (0.63)	3.08 (0.62)	3.15 (0.63)
Care needs level	1.74 (0.72)	1.89 (0.85)	1.82 (0.79)
No (%) female	262 (74)	240 (73)	502 (74)
Age (years)	84.1 (7.2)	84.4 (7.2)	84.3 (7.2)
No (%) widowed	235 (67)	217 (66)	452 (66)
No (%) with enough money	195 (55)	184 (56)	379 (56)
No (%) publicly funded	199 (56)	197 (60)	396 (58)
Total No of diagnoses	5.0 (2.2)	4.8 (2.2)	4.9 (2.2)
Total No of drugs	7.2 (3.1)	7.2 (3.3)	7.2 (3.2)
No (%) antidepressants	133 (38)	102 (31)	235 (34)
No (%) benzodiazepines	105 (30)	97 (29)	202 (30)
No (%) depression (GDS >4*)	117/317 (37)	98/297 (33)	215/614 (35)
AMTS score†	7.1 (2.4)	7.4 (2.3)	7.2 (2.4)
Systolic blood pressure (mm Hg)	148 (28)	148 (28)	148 (28)
Diastolic blood pressure (mm Hg)	74 (13)	75 (14)	74 (13)
**Outcomes**			
LLFDI total disability†	37.5 (5.9)	37.9 (6.8)	37.7 (6.3)
LLFDI total function†	45.7 (10.5)	45.1 (10.4)	45.4 (10.4)
Timed up and go (seconds)	31 (24)	30 (21)	31 (22)
Elderly mobility scale score†	15.7 (4.0)	16.1 (3.4)	15.9 (3.7)
Balance (1-3)	2.0 (1.4)	2.2 (1.4)	2.1 (1.4)
Seconds on one leg	1.0 (2.6)	1.3 (3.3)	1.2 (3.0)
LSI-Z score†	15.4 (5.1)	15.9 (5.1)	15.6 (5.1)
Quality of life (visual analogue scale score)	72.4 (20.5)	70.2 (20.8)	71.4 (20.7)
Previous falls/1000 bed days	3.9 (14)	1.7 (9)	3.9 (12)

AMTS=abbreviated mental test score; GDS=geriatric depression scale; LLFDI=late life function and disability instrument; LSI-Z=life satisfaction index.

*Higher score=greater depression.

†Higher score=better.

We found no evidence of interactions between depression, group status, and time for any of the primary outcomes. Therefore, all analyses include residents with and without depression. No significant interaction existed for the disability overall and limitation overall scales of the late life function and disability instrument (table 2).

**Table 2 tbl2:** Effect of an activity intervention on late life function and disability instrument disability and limitation scales. Values are mean (SE)

Disability overall:				0.25*
Activity group	36.3 (0.6)	−1.3 (0.6)	35.4 (0.6)	
Social group	35.9 (0.5)	−1.6 (0.4)	34.5 (0.6)	
Limitation overall:				0.27
Activity group	58.8 (1.4)	−8.3 (1.9)	49.8 (1.5)	
Social group	57.8 (1.3)	−7.3 (2.0)	50.1 (1.4)	

*For interaction between time and intervention group from repeated measures analysis comparing groups over time; no interaction between cognition and intervention.

A significant interaction existed between cognition and group status for the function overall scale of the late life function and disability instrument (P=0.024), and we report the results separately for this variable. For residents with normal cognition (abbreviated mental test score ≥7) the activity group deteriorated less in overall function (late life function and disability instrument, total function component score) in the first six months of follow-up (table 3). For those with impaired cognition (abbreviated mental test score <7), the activity intervention had no significant effect on function and the mean scores for activity group participants tended to deteriorate faster than those of the social group. A similar significant interaction (P=0.015) in the lower limb subscale score of the late life function and disability instrument showed a maintenance in score in the activity group for those with normal cognition (intervention group score 48.8 at baseline, 48.1 at six months, and 47.7 at 12 months; control group 49.5, 45.9, and 46.5) but no differences in the cognitively impaired subgroup. Other subscale scores, including advanced lower limb, upper limb, overall disability, and social and personal role subscales on the late life function and disability instrument; mobility and balance measures; quality of life; and time to fall, were not affected by the activity intervention (tables 2 and 4).

**Table 3 tbl3:** Effect of an activity intervention on function, mobility and balance, and quality of life. Values are mean (SE)

Measure and group	Baseline	Change 0-6 months	6 months	Change 6-12 months	12 months	P value
**Function overall (LLFDI)**						
Cognition normal:						0.024*
Activity group	44.5 (1.0)	−0.8 (0.5)	43.9 (1.0)	−0.2 (0.3)	43.6 (1.0)	
Social group	44.9 (1.0)	−2.2 (0.6)	42.7 (1.0)	0.3 (0.7)	43.0 (1.0)	
Cognition impaired:						
Activity group	46.7 (1.4)	−2.1 (1.1)	44.4 (1.5)	−2.8 (1.1)	42.5 (1.7)	
Social control	47.1 (1.2)	−0.3 (1.4)	47.1 (1.3)	0.2 (1.0)	46.8 (1.4)	
**Mobility and balance**						
TUG (seconds):						0.67
Activity group	35.4 (2.1)	3.0 (1.5)	38.9 (2.2)	0.3 (1.4)	38.5 (2.3)	
Social group	34.6 (2.0)	2.4 (1.0)	36.9 (2.1)	1.2 (0.5)	37.0 (2.1)	
EMS (% score ≤16):		(% increased:% decreased)		(% increased:% decreased)		0.48
Activity group	47.0 (4.01)	9:11	45.8 (4.2)	5:8	43.5 (4.4)	
Social group	46.0 (3.9)	9:8	49.2 (4.0)	5:9	43.9 (4.1)	
Balance (% ≥10 seconds tandem stance):		(% increased:% decreased)		(% increased:% decreased)		0.16
Activity group	42.9 (4.0)	14:13	42.4 (4.4)	10:13	38.9 (4.7)	
Social group	38.2 (3.8)	17:11	47.0 (4.1)	7:17	34.8 (4.3)	
**Quality of life**						
EuroQol (score/12):						0.49
Activity group	9.7 (0.2)	0.2 (0.1)	9.9 (0.2)	0.2 (0.2)	10.0 (0.2)	
Social group	9.6 (0.1)	0.3 (0.2)	10.0 (0.2)	0.2 (0.2)	10.1 (0.2)	
LSI-Z (score/20):						0.35
Activity group	15.3 (0.4)	−0.4 (0.2)	14.7 (0.4)	−0.3 (0.2)	14.5 (0.4)	
Social group	14.9 (0.4)	−0.3 (0.3)	14.6 (0.4)	0.3 (0.3)	14.9 (0.4)	

*For interaction between time, cognition, and intervention group from repeated measures analysis.

EMS=elderly mobility scale (higher scores=better function); LLFDI=late life function and disability instruments; LSI-Z=life satisfaction index; TUG=timed up and go test.

**Table 4 tbl4:** Effect of an activity intervention on falls in elderly people in residential care

Group	No (%) who fell	Median (range) time to first fall (days)	Hazard ratio (95% CI)	P value
Activity group	162/310 (52)	217 (2-411)	1.1 (0.84 to 1.44)	0.48
Social group	146/329 (44)	177 (1-404)		

A significant interaction existed between cognition, group status, and time for depression as a secondary outcome. More people with impaired cognition in the activity group had pronounced depressive symptoms after the activity intervention than did those with cognitive impairment in the social group (table 5). We did not analyse fear of falling, as at least 40% of the sample had incomplete data for this measure because the residents struggled to allocate a number to the specific fear of falling task. No difference existed in the proportion of residents who were either admitted into higher level care or died during the 12 months of the trial between the activity group (55/330, 17%) and the social group (47/352, 13%) (P=0.61). We found no evidence of a difference in the rate of hospital admissions between the social group and the activity group (P=0.55) (table 6), nor any difference in the level of adverse outcomes (P=0.75).

**Table 5 tbl5:** Effect of activity intervention on depression and aches and pains (secondary outcome measures) in older people in residential care

Secondary outcomes and adverse events	Baseline	6 months	12 months	P value
**Geriatric depression scale score >4 (% (SD))**				
Cognitively impaired:				0.004*
Activity group	41.9 (7.1)	48.5 (7.7)	63.8 (8.0)	
Social group	38.4 (6.1)	35.2 (6.2)	35.9 (6.6)	
Cognitively normal:				
Activity group	37.1 (3.7)	37.5 (3.8)	37.9 (4.1)	
Social group	44.5 (3.8)	46.8 (4.0)	49.0 (4.2)	
**Aches and pains (% (95% CI))**				
Activity group	48.8 (40.4 to 54.9)	46.7 (39.3 to 54.9)	42.4 (34.7 to 50.4)	0.75†
Social group	49.5 (42.6 to 56.5)	51.1 (43.8 to 58.4)	48.8 (41.2 to 56.6)	

*P value for interaction between time, cognition, and intervention group from repeated measures analysis.

†P value for interaction between time and intervention group from repeated measures.

**Table 6 tbl6:** Effect of activity intervention on hospital admissions (secondary outcome measure) in older people in residential care

Group	Admitted at least once (%)	Mean (SD) length of stay (days)	Incidence rate ratio (95% CI)
Activity group	103/330 (31)	6.94 (7.78)	0.91 (0.65 to 1.25), P=0.55
Social group	120/352 (34)	6.12 (6.22)	

When we considered uptake of the intervention, analyses found that those who achieved their goal (187/330, 57%) were no more likely to have an improvement in function than those who did not (P=0.84). We were unable to accurately ascertain to what degree the promoting independence plan was adhered to. Compliance with the intervention may have been low. Checklists of activity sessions were filled in less than half of the time, and intervention nurses’ reports of compliance were usually based on the first three months of the trial and were completed for only two thirds of intervention group residents. Intervention nurses reported that 145 (44%) of the intervention group residents did few or no activity sessions. We found no greater change in outcomes for those with greater compliance.

## Discussion

An activity programme had no impact on overall function for elderly people in residential care. The difference between the activity group and social group in residents with normal cognition was of questionable clinical significance, and no changes occurred in observed function, quality of life, or falls. Neither achievement of goals nor compliance made any difference to improvement in function. Residents with impaired cognition showed no maintenance of function and may have become more depressed.

This trial tested the effectiveness of the intervention in a representative group of homes and followed a successful efficacy trial.^21^ Other trials showing greater levels of success in changing quality of life or performance based assessments of mobility have tested more vigorous and resource intensive interventions.^6^ ^7^ ^9^ ^38^ This result highlights the importance of specificity of training. The training approach in this study focused on practising overall functional tasks embedded within daily activities, facilitated by existing staff. Unlike other studies, it did not use an exercise focused approach to intensively work on underlying impairments such as muscle weakness or balance problems. As a result, the fact that the change seen was small and in overall function without change in balance or mobility performance is probably not surprising. This intervention also did not consider any environmental barriers or psychological factors that might need to be tackled to facilitate changes at the level of disability or quality of life. The social group may have had improved quality of life as a result of the social visits. In an institutional setting with fixed environmental constraints, changes in disability will probably be limited compared with changes in function.

Residents with poor cognition did not benefit from this intervention, and their mood may have been adversely affected. Goal setting interventions may require normal cognition, as goal oriented interventions need to be owned by the individual person. The use of client led goal setting interventions for those with poor cognition should be approached with caution, and further refinement in potential interventions is needed for those with poor cognition.

### Strengths of the trial

The trial design, including randomisation, blinded outcome ascertainment, and the adjusted analyses, was robust. The results can be considered generalisable, as more than 80% of homes and participants invited to participate did so.

Internal contamination is unlikely, as homes were separated and cross over of staff between homes was rare. The control group received social visits, a discussion of their activities, and a summary, controlling for the time spent by the gerontology nurse with participants in the activity group. The differential effect seen for residents with and without normal cognition highlights the need for adequately powered randomised trials to examine significant interactions and carry out in-depth subgroup analyses for which this study was not powered

### Uptake of the intervention

Almost all (93%) residents were visited, three quarters were able to set a goal, and three quarters of these were able to achieve that goal. Although we could not accurately ascertain how well the intervention was taken up or sustained, participation in the activities was estimated to be low (45% doing none or few). Anecdotal reports from the intervention nurses indicated that the staff uptake was variable; some homes had excellent buy-in, whereas in others staff participation was less obvious. Organisational change is often needed to effectively implement long term changes in health care.^39^ Either a more intensive intervention or more effort in implementation would be needed to achieve functional improvement in this population.

### Conclusions

An activity programme based on usual activities of daily living, targeted to a personal goal that is meaningful to an individual resident, did not help to preserve physical function in frail elderly people with normal cognition in residential care and may have adversely affected those with poor cognition. Low compliance with activity recommendations was likely. To be successful, such interventions may need a higher intensity of activity and more effective reinforcement by care workers. Interventions should be carefully targeted to those people likely to engage and respond within the residential care context.

**What is already known on this topic**

Improving activity levels for elderly people in residential care can improve quality of life but may increase falls
Sustainable ways to improve activity in residential care have not been identified
Few trials have tested functionally based activity programmes in residential care

**What this study adds**

A goal oriented programme based on activities of daily living made no real impression on function or falls in elderly people in long term residential care
For those with impaired cognition this intervention was not helpful and may have increased depressive symptoms

## Notes

We thank the participants who contributed so eagerly and diligently to this research, and we are grateful to the staff of the participating residential care facilities. We also thank our research team Linda Venables, Gaye Purton, and Helen Roud and the many others who contributed.

Contributors: All the authors contributed to the study design or protocol design, or both, interpreted the data, and wrote the paper. NK obtained funding, directed the project, and wrote the paper. ER advised on data management and did the analysis. KP was the study coordinator. MvR managed the data. MP and KP conceived the intervention and wrote the paper. LK was study administrator and contributed to data collection, data analysis, and manuscript preparation. NK is the guarantor.

Ethical approval: The Auckland Ethics Committee, now named the Multi-regional Ethics Committee, approved the study in 2004.

Provenance and peer review: Not commissioned; externally peer reviewed.

## Notes

**Cite this as: ***BMJ* 2008;337:a1445
